# Supplementary material for: Assessment of segregation variance estimates from derivation, simulations, and empirical data in autotetraploid species exemplified in potato
Source: Genetics. 2026 Feb 6;232(4):iyag031. doi: 10.1093/genetics/iyag031 (PMC13050197; doi:10.1093/genetics/iyag031)
Supplement: iyag031_Supplementary_Data [file iyag031_supplementary_data.zip › Supplemental_Material_GENETICS-2025-308796.pdf]

Assessment of segregation variance estimates from derivation, simulations, and empirical data in autotetraploid species exemplified in potato

Po-Ya Wu[1,2], Kathrin Thelen[1,3], Stefanie Hartje[4], Katja Muders[5], Vanessa Prigge[6], Benjamin Stich[1,3], and Delphine Van Inghelandt[1,2,*]

[1]Federal Research Centre for Cultivated Plants (JKI), Institute for Breeding Research on Agricultural Crops, 18190 Sanitz, Germany
[2]Leibniz Institute of Plant Genetics and Crop Plant Research (IPK), 06466 Seeland, Germany
[3]Utilization of Plant Genetic Resources for Breeding Purposes, Faculty of Agricultural and Environmental Sciences, University of Rostock, 18059 Rostock, Germany
[4]Europlant Innovation GmbH & Co. KG, 21337 Lüneburg, Germany
[5]NORIKA GmbH, 18190 Sanitz, Germany
[6]SaKa Pflanzenzucht GmbH & Co. KG, 24340 Windeby, Germany
[*]Corresponding author: Delphine Van Inghelandt, Delphine.Inghelandt@julius-kuehn.de

January 28, 2026

SUPPLEMENTARY MATERIAL

Method S1

Derivation of segregation variance considering double reduction (DR)

We denote that a genotype at two linked loci, A and B, can be expressed as $A_1B_1/A_2B_2/A_3B_3/A_4B_4$ (see Figure S1). Only bi-allelic loci are considered in our study, and, thus, any A_h and B_h is coded as 0 or 1 for representing the reference or the alternative allele, respectively, where h indicates the 1, 2, 3, or 4th homolog. Similarly to the derivation of equation [3], we assume that phased parental haplotypes and the recombination rate (c) between two loci A and B are known, and only consider additive effects. Therefore, $cov(x_{\mathrm{A}}, x_{\mathrm{B}})$ can be derived based on the linkage disequilibrium (LD) between pairs of loci among all possible combinations of genotypes produced by a set of two parents. Differently from the derivation of equation [3], we follow the assumption of complete quadrivalent pairing among homologous chromosomes during meiosis (Luo et al., 2004). Further, the order of the loci locations on the genetic map is assumed to be centromere, locus A, and locus B. The rate of double reduction at locus A is α_A, and the double reduction rate at loci B can be expressed in function of α_A and c (Luo et al., 2004). Fisher (1947) classified the gametes at two linked loci for each genotype into 11 modes of gamete formation, and Luo et al. (2004) carefully calculated the probabilities of each mode (see Table S4, modified from Table 1 of Luo et al. 2004), where the 11 modes of gamete formation result in 136 distinct gametes for each genotype (Table S5). Therefore, the probability of each gamete is a function of c and α_A.

When two parents are crossed, 18,496 ($= 136^2$) genotypes are possible at two loci

in the F1 progenies. All possible genotypes and their corresponding probabilities are shown in Table S6. To establish covariance between the two linked loci, $cov(x_{\mathrm{A}}, x_{\mathrm{B}})$, we follow the definition of covariance like equation [2] by using each genotype's expression and its probability. Consequently, the covariance between the two loci can be shortened to:

$$cov(x_{\mathrm{A}}, x_{\mathrm{B}}) = \{\frac{1}{3}\sum_{h=1}^{4}(\mathrm{A}_h^{\mathrm{P1}}\mathrm{B}_h^{\mathrm{P1}}+\mathrm{A}_h^{P2}\mathrm{B}_h^{\mathrm{P2}})-\frac{1}{12}(x_{\mathrm{A}}^{\mathrm{P1}}x_{\mathrm{B}}^{\mathrm{P1}}+x_{\mathrm{A}}^{\mathrm{P2}}x_{\mathrm{B}}^{\mathrm{P2}})\}\cdot(1-\frac{4}{3}c)(1+2\alpha_A),$$

where $\mathrm{A}_h^{\mathrm{P1}}$ and $\mathrm{B}_h^{\mathrm{P1}}$ ($\mathrm{A}_h^{\mathrm{P2}}$ and $\mathrm{B}_h^{\mathrm{P2}}$) denote the genotypic indicator (0/1 for the reference/alternative allele, respectively) at the hth homolog of A and B loci for P1 (P2), and $x_{\mathrm{A}}^{\mathrm{P1}}$ and $x_{\mathrm{B}}^{\mathrm{P1}}$ ($x_{\mathrm{A}}^{\mathrm{P2}}$ and $x_{\mathrm{B}}^{\mathrm{P2}}$) the genotypic indicator of A and B loci for P1 (P2) ranging from 0 to 4, where $x_{\mathrm{A}}^{\mathrm{P1(2)}} = \sum_{h=1}^{4}\mathrm{A}_h^{\mathrm{P1(2)}}$ and $x_{\mathrm{B}}^{\mathrm{P1(2)}} = \sum_{h=1}^{4}\mathrm{B}_h^{\mathrm{P1(2)}}$.

REFERENCES

Fisher, R. A. (1947). The theory of linkage in polysomic inheritance. *Philosophical Transactions of the Royal Society of London. Series B, Biological Sciences*, 233(594):55–87.

Gallais, A. (2003). *Quantitative genetics and breeding methods in autopolyploid plants.* Institut national de la recherche agronomique.

Luo, Z. W., Zhang, R. M., and Kearsey, M. J. (2004). Theoretical basis for genetic linkage analysis in autotetraploid species. *Proceedings of the National Academy of Sciences of the United States of America*, 101(18):7040–7045.

Tables

Table S1: The complete list of the 1296 possible genotypes in a F1 family of a bi-parental cross in case of bivalent formation during meiosis and their corresponding probabilities, depending on the parental gametes and their probabilities. c is the recombination rate between loci A and B.

File: Table_S1_all_genotype_1296_combination.xlsx

Table S2: Description of the 15 environments (i.e., year-location combinations) that were used in the experiment and their respective properties. The breeding companies are Europlant (EUROPLANT Innovation GmbH & Co. KG), Norika (NORIKA GmbH), and SaKa (SaKa Pflanzenzucht GmbH & Co. KG).

Environment	No. of entries	No. of families	No. of blocks	No. of plants per plot
Europlant 2019 Kaltenberg	299	46	4	10
Europlant 2020 Kaltenberg	297	47	4	16
Europlant 2020 Böhlendorf	287	46	2	16
Europlant 2021 Kaltenberg	300	47	4	16
Europlant 2021 Böhlendorf	300	47	1	16
Norika 2019 Groß Lüsewitz	300	17	2	9
Norika 2020 Groß Lüsewitz	300	17	4	18
Norika 2020 Mehringen	300	17	3	20
Norika 2021 Groß Lüsewitz	297	17	4	18
Norika 2021 Mehringen	300	17	2	20
Saka 2019 Windeby	458	107	8	10
Saka 2020 Windeby	387	99	8	16
Saka 2020 Gransebieth	387	99	8	16
Saka 2021 Windeby	387	99	8	16
Saka 2021 Gransebieth	387	99	8	16

Table S3: Medians of Pearson correlation coefficient (PCC) and root mean square deviations (RMSD) between segregation variance predicted from the algebraic derivations with ($\sigma^2_{G(\text{eq}-\text{phased})}$) and without ($\sigma^2_{G(\text{eq}-\text{unphased})}$) phased parental haplotypes using different number of families (n_{cross} = 10, 50, 100, 250, and 500) among 30 simulation runs.

	n_{cross}				
	10	50	100	250	500
PCC	0.226	0.268	0.259	0.266	0.271
RMSD	13.831	14.078	14.108	14.295	13.964

Table S4: The probability of the 11 modes of gamete formation from a genotype, $A_1B_1/A_2B_2/A_3B_3/A_4B_4$, in a scenario with two loci A and B in case of complete quadrivalent pairing during meiosis. c is the recombination rate between loci A and B. α_A is the rate of double reduction at locus A (adapted from Table 1 of Luo et al. (2004)).

Mode	Gamete $(1 \le i, j, k, l \le 4)$	Frequency	Double reduction occurs at locus	The number of recombination events	Probability
1	A_iB_i/A_iB_i	4	A,B	0	$\frac{27\cdot\alpha_A\cdot(1-c)^2}{108}$
2	A_iB_j/A_iB_j	12	A,B	2	$\frac{3\cdot\alpha_A\cdot c^2}{108}$
3	A_iB_i/A_iB_j	12	A	1	$\frac{18\cdot\alpha_A\cdot c\cdot(1-c)}{108}$
4	A_iB_j/A_iB_k	12	A	2	$\frac{6\cdot\alpha_A\cdot c^2}{108}$
5	A_iB_i/A_jB_i	12	B	1	$\frac{6\cdot(1-\alpha_A)\cdot c\cdot(1-c)}{108}$
6	A_iB_j/A_kB_j	12	B	2	$\frac{2\cdot(1-\alpha_A)\cdot c^2}{108}$
7	A_iB_i/A_jB_j	6	—	0	$\frac{18\cdot(1-\alpha_A)\cdot(1-c)^2}{108}$
8	A_iB_i/A_jB_k	24	—	1	$\frac{6\cdot(1-\alpha_A)\cdot c\cdot(1-c)}{108}$
9	A_iB_j/A_jB_i	6	—	2	$\frac{2\cdot(1-\alpha_A)\cdot c^2}{108}$
10	A_iB_j/A_jB_k	24	—	2	$\frac{2\cdot(1-\alpha_A)\cdot c^2}{108}$
11	A_iB_j/A_kB_l	12	—	2	$\frac{2\cdot(1-\alpha_A)\cdot c^2}{108}$

—: represents that no double reduction occurs at both loci A and B.

Table S5: The probabilities of the 136 gametes from a genotype, $A_1B_1/A_2B_2/A_3B_3/A_4B_4$, in a scenario with two loci A and B in case of complete quadrivalent pairing during meiosis. c is the recombination rate between loci A and B. α_A is the rate of double reduction at locus A.

File: Table_S5_prob_136_gametes_quadrivalent.xlsx

Table S6: The complete list of the 18496 (136^2) possible genotypes in a F1 family of a bi-parental cross in case of complete quadrivalent pairing during the meiosis and their corresponding probabilities, depending on the parental gametes and their corresponding probabilities. c is the recombination rate between loci A and B. α_A is the rate of double reduction at locus A.

File: Table_S6_all_genotypes_136x136_combination_quadrivalent.xlsx

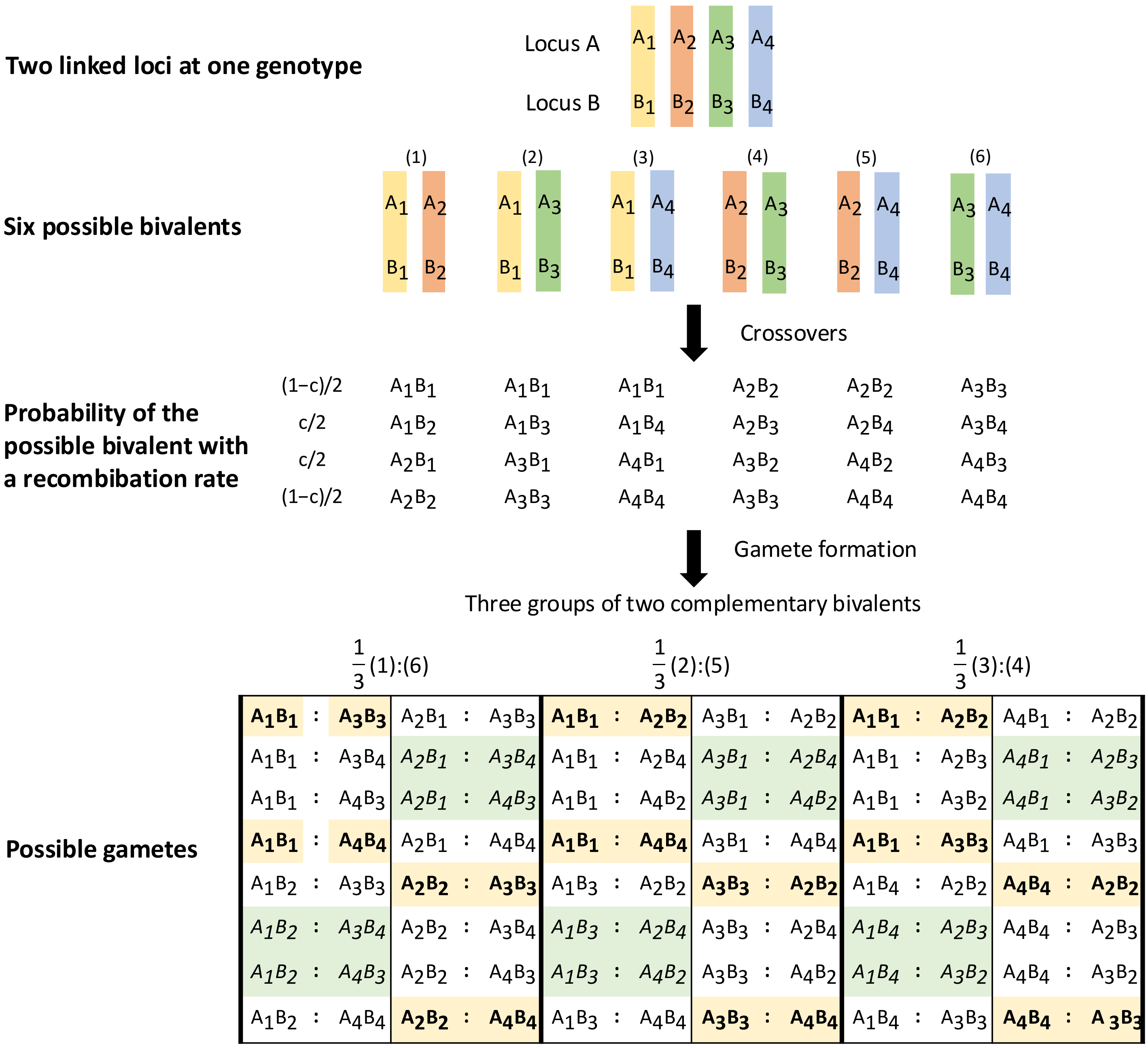

Figure S1: Formation of the gametes from a autotetraploid genotype (considering only random bivalents during meiosis). A genotype at two linked loci, A and B, with a frequency of recombination c across four homologs are expressed $A_1B_1/A_2B_2/A_3B_3/A_4B_4$. Ignoring linkage phases (coupling and repulsion), there are 16 types of gametes for each pair of groups but in total there are 36 types of gametes instead of 48. This is because both gametes with parental associations (in bold) and those with two recombined chromosomes (in underline) are present twice. This figure is revised from Figure 1.4 in Gallais (2003).

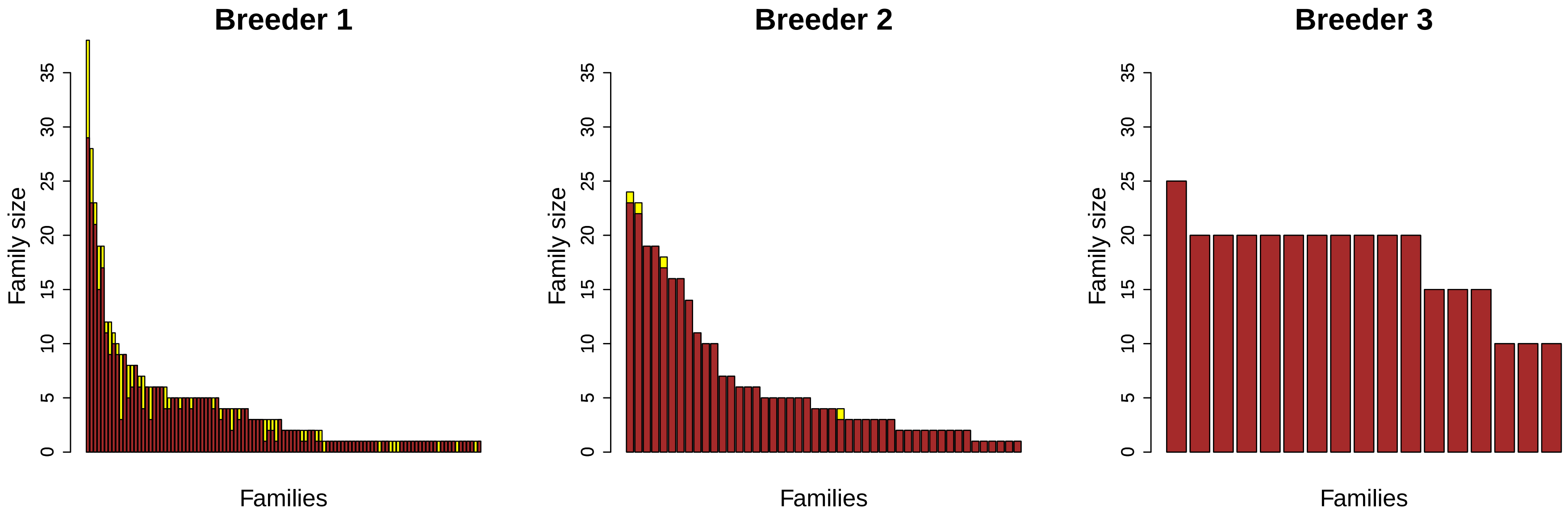

Figure S2: The distribution of the family size of the 171 families across the three breeding companies at A clone stage, respectively. The bar filled in yellow represented the 171 families among the 1058 phenotyped entries and the bar in brown the 163 families among the 980 genotyped entries.

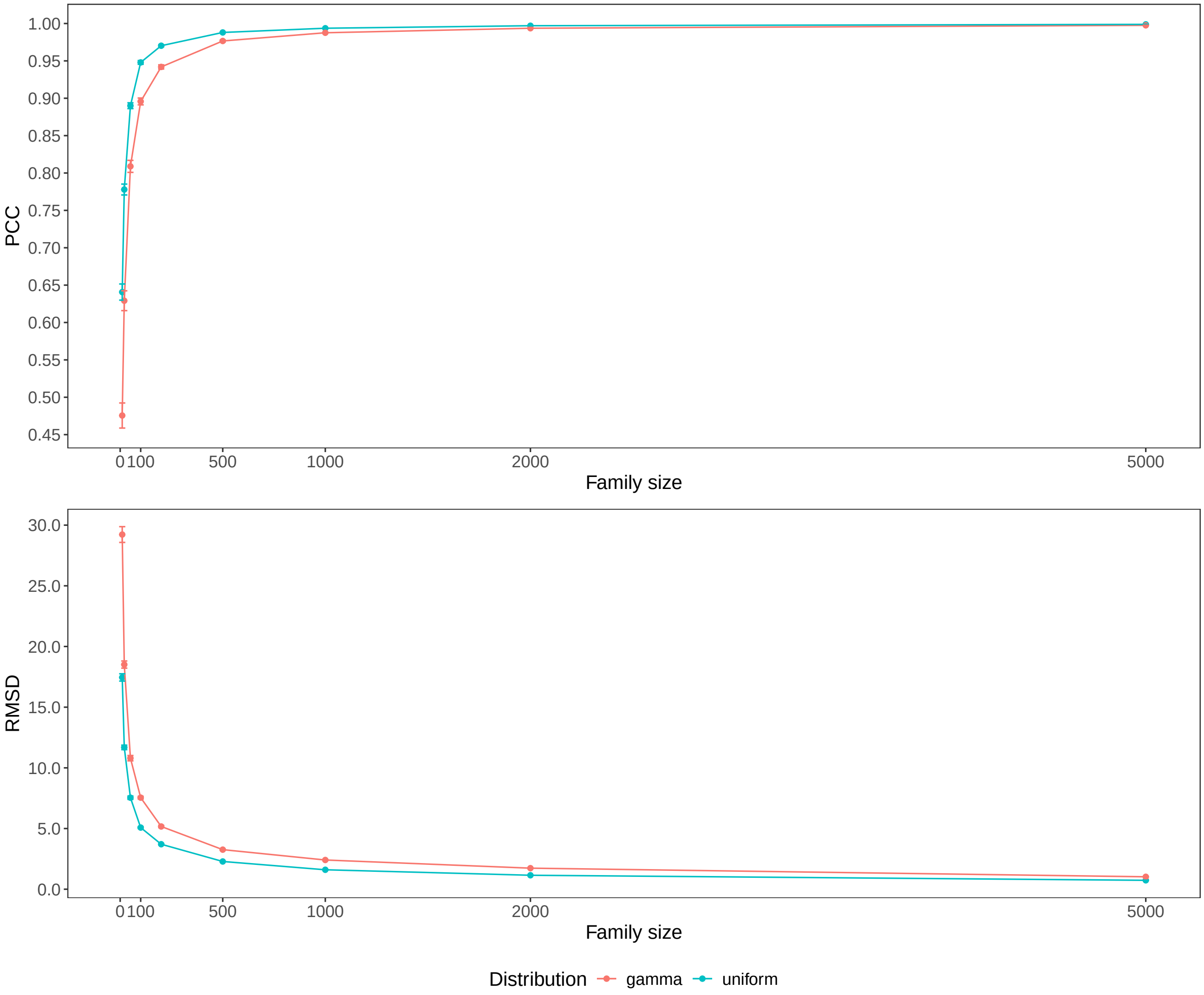

Figure S3: Plot of median of Pearson correlation coefficient (PCC, upper) and root mean square deviation (RMSD, bottom) across the 30 runs against different family sizes. The error bar is the standard error of PCC and RMSD across the 30 runs. The PCC and RMSD were calculated between $\sigma^2_{G(\mathrm{n_{cross=100}},\mathrm{distr},n_{\mathrm{p/cross}},\mathrm{M_1})}$ and $\sigma^2_{G(\mathrm{eq-phased})}$. $\sigma^2_{G(\mathrm{n_{cross=100}},\mathrm{distr},n_{\mathrm{p/cross}},\mathrm{M_1})}$: the segregation variance obtained from the summation of sample variance per chromosome; $\sigma^2_{G(\mathrm{eq-phased})}$: the segregation variance estimated by the derived formula with phased parental haplotypes; n_{cross}: the number of families (100 here); distr: the distribution of the families size across families: uniform or gamma distribution; and $n_{\mathrm{p/cross}}$: the family size.

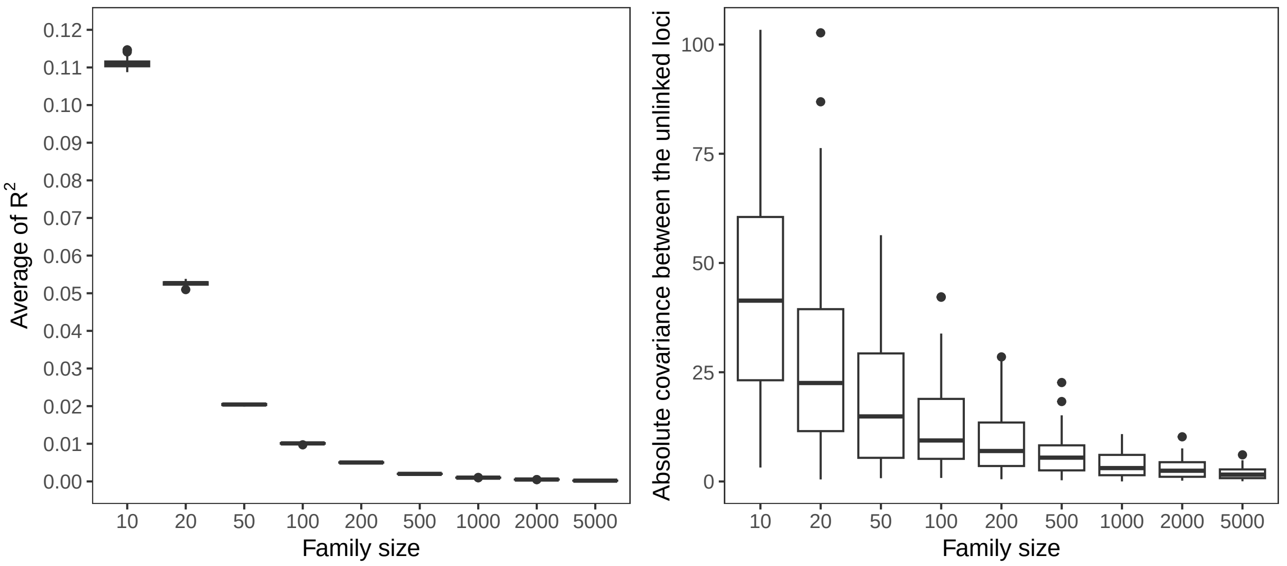

Figure S4: Boxplot of the average of R^2 among all pairwise unlinked loci (left), and the absolute total covariance between the unlinked loci (right), against different family sizes across 50 families. R^2 was calculated as the square of the Pearson correlation coefficient between two unlinked loci among the simulated progenies.

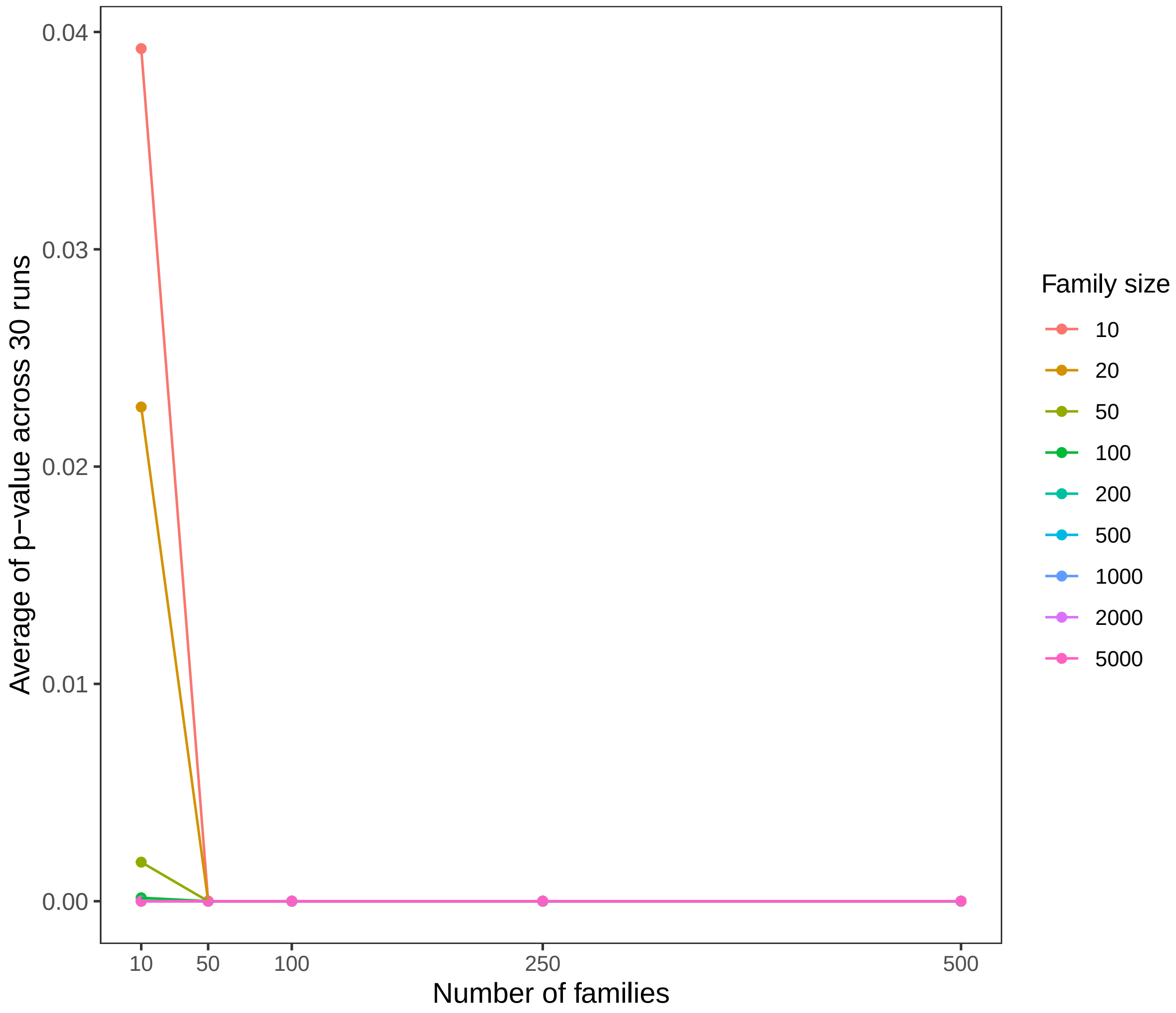

Figure S5: The average of the p−value of the Pearson correlation coefficient between the segregation variance from the derived formula ($\sigma^2_{G(\mathrm{eq-phased})}$) and the one estimated from simulated progenies using different numbers of families and family sizes and ignoring the covariance between the unlinked loci ($\sigma^2_{G(n_{\mathrm{cross}},distr,n_{\mathrm{p/cross}},\mathrm{M}_1)}$) among 30 simulation runs.

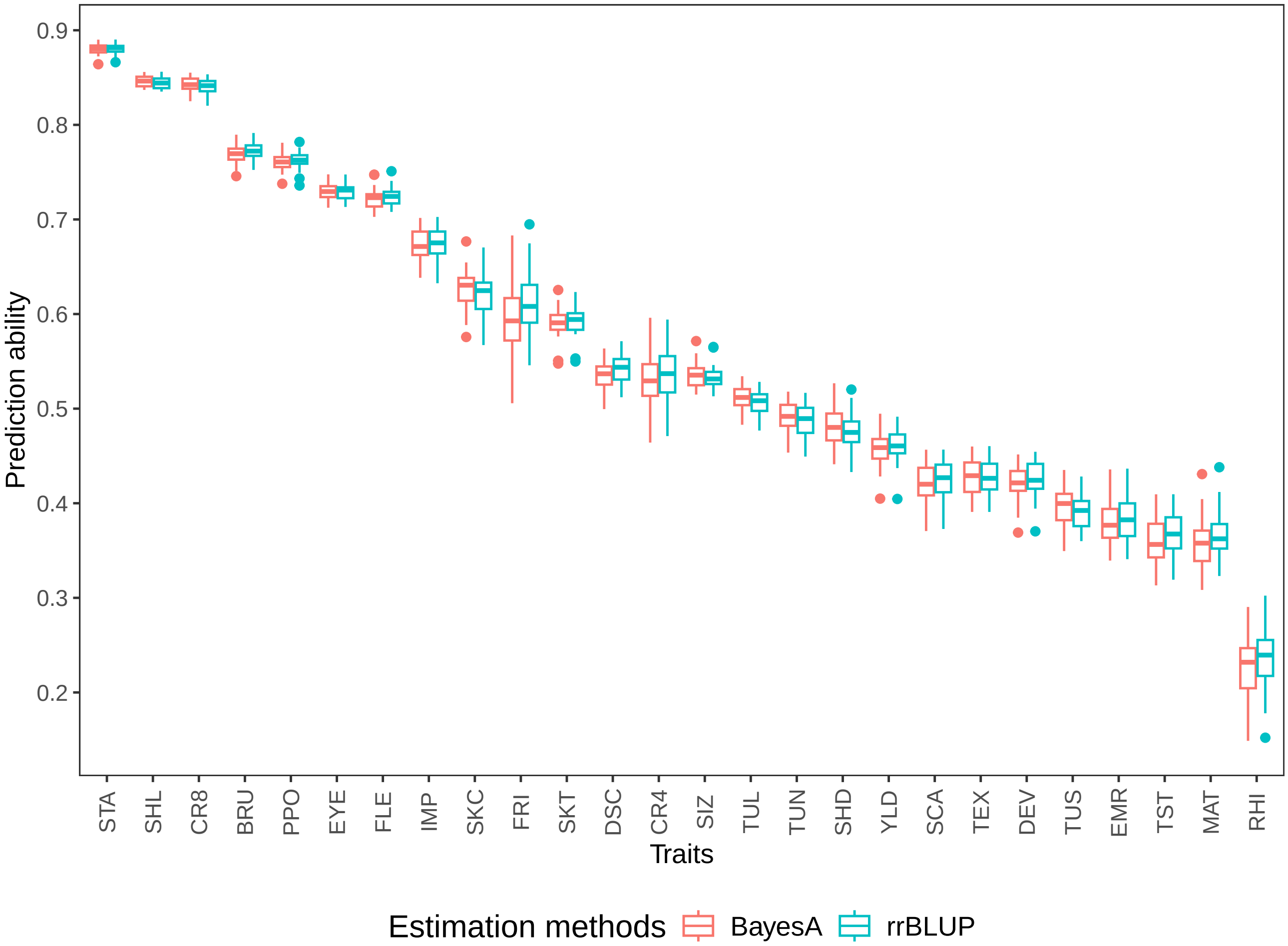

Figure S6: Boxplots of prediction abilities for all the assessed traits based on 988 clones and 25602 markers using rrBLUP and Bayes A estimations across 30 five-fold cross-validation (CV) runs. The prediction ability was calculated as the median of the correlation between the adjusted entry means and the estimated genetic values from the genomic prediction model among the five-fold CV within each replicate.
